# Supplementary material for: Propionic acid toxicity and utilization of α-ketobutyric acid in Neisseria meningitidis via the methylcitrate cycle under specific conditions
Source: Microbiol Spectr. 2025 Oct 20;13(12):e00783-25. doi: 10.1128/spectrum.00783-25 (PMC12671195; doi:10.1128/spectrum.00783-25)
Supplement: Supplemental Material — Fig. S1 to S10 and legends for Files S1 to S6. [file spectrum.00783-25-s0007.pdf]

Propionic Acid Toxicity and Utilization of α-Ketobutyric Acid in *Neisseria meningitidis* via the Methylcitrate Cycle Under Specific Conditions

Adelfia Talà [a], Matteo Calcagnile [b], Silvia Caterina Resta [b], Salvatore Maurizio Tredici [b], Giuseppe Egidio De Benedetto [c], Cecilia Bucci [b], Pietro Alifano [b,*]

[a] *Department of Biological and Environmental Sciences and Technologies, University of Salento, Via Monteroni 165, Lecce 73100, Italy.*

[b] *Department of Experimental Medicine, University of Salento, Via Monteroni 165, Lecce 73100, Italy.*

[c] *Laboratory of Analytical and Isotopic Mass Spectrometry, Department of Cultural Heritage, University of Salento, Via Monteroni 165, Lecce 73100, Italy*

*Corresponding author: pietro.alifano@unisalento.it

RUNNING TITLE: Meningococcal propionate and α-ketobutyrate metabolism

SUPPLEMENTARY MATERIAL

Supplementary Figures

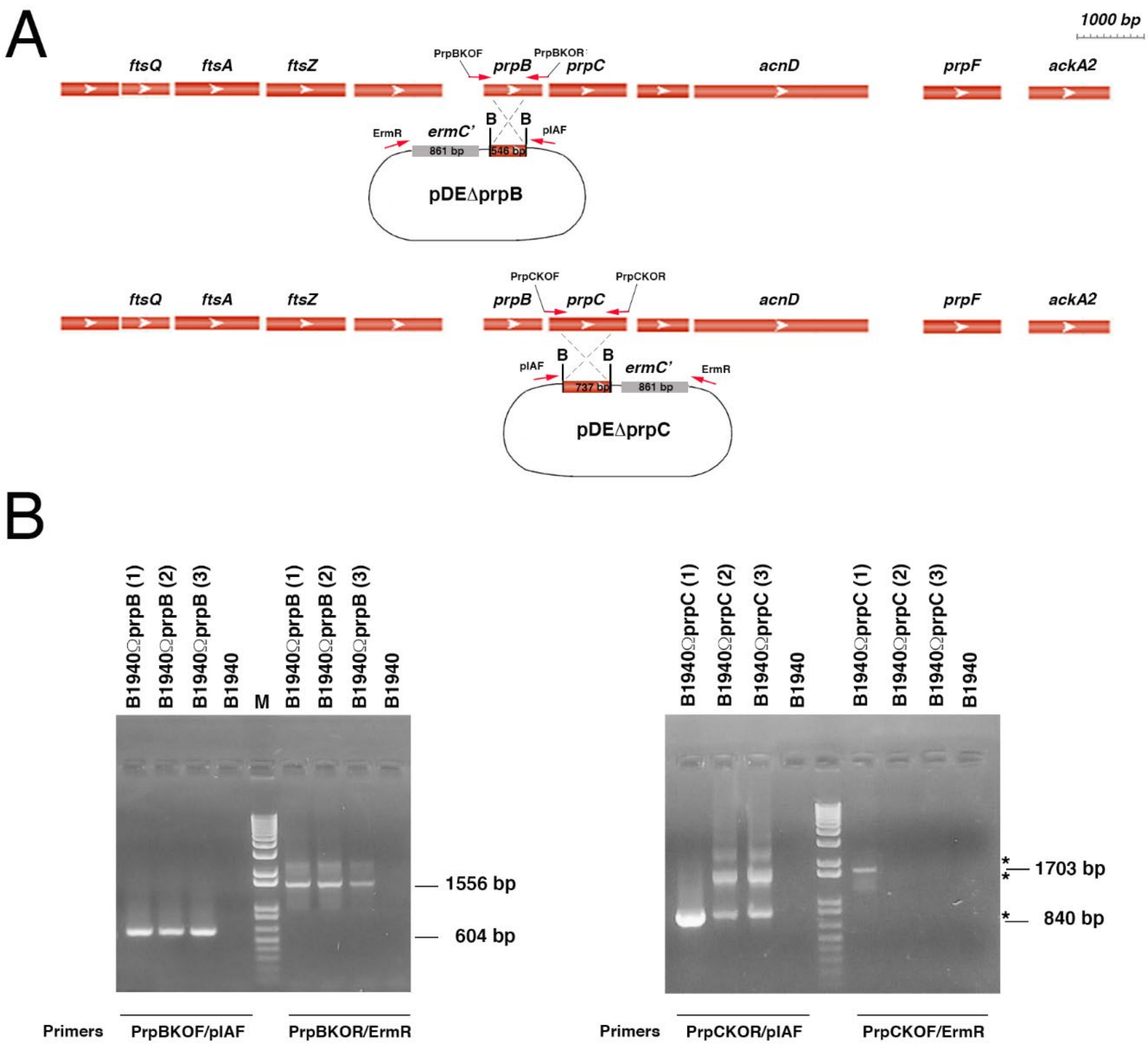

Fig. S1. Genetic inactivation of the methylcitrate *prpB* and *prpC* genes in *N. meningitidis* B1940. **A.** Map of the genomic region encompassing the MCC gene cluster in *N. meningitidis* MC58 and strategy for insertional inactivation of *prpB* and *prpC*. **B.** PCR analysis by agarose gel confirmed the insertion of the erythromycin-resistant circular cassette by a single cross-over event in *prpB* or *prpC*. For *prpB* inactivation, three recombinant clones were obtained. As a result of the recombination event, two DNA fragments of the expected sizes of 604 bp and 1556 bp were amplified in the transformed strains B1940ΩprpB(1), B1940ΩprpB(2) and B1940ΩprpB(3) using the primer pairs PrpBKOF/pIAF and PrpBKOR/ErmR, respectively (shown in **A**), according to the orientation of the fragment in the shuttle vector pDEX. For *prpC* inactivation, a single recombinant clone, B1940ΩprpC(1), was obtained. As a result of the recombination event, two DNA fragments of the expected sizes of 840 bp and 1703 bp were amplified in this transformed strain using the primer pairs PrpCKOR/pIAF and PrpCKOF/ErmR, respectively (shown in **A**), according to the orientation of the fragment in the shuttle vector pDEX. The other two transformants analyzed (B1940ΩprpC(2) and B1940ΩprpC(3)) gave either spurious or absent PCR products.

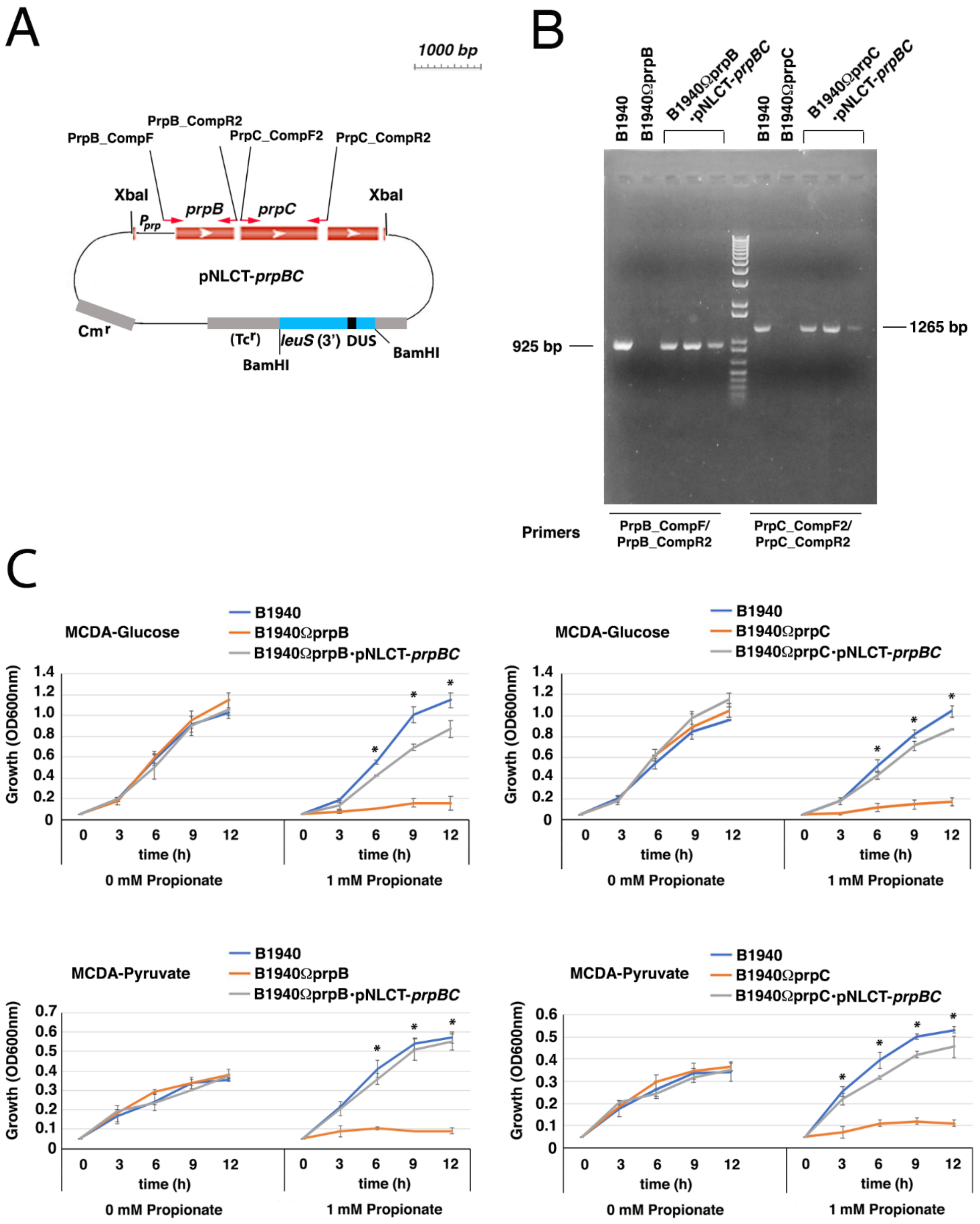

Fig. S2. Complementation of *prpB* and *prpC* mutants and growth curves of complemented *prpB* and *prpC* mutants. **A.** Genetic map of the integrative plasmid pNLCT-*prpBC*. Cm^r, chloramphenicol-resistance genetic determinant; *leuS* (3'), 3'-end of *leuS* gene for integration in *N. meningitidis* chromosome; DUS, DNA uptake sequence. The *leuS*-DUS region was inserted into the BamHI site of the tetracycline-resistance genetic determinant (Tc^r). The *prp* operon region comprising the *Pprp* promoter was inserted into the XbaI site of the vector plasmid pNLCT1 generating the plasmid pNLCT-*prpBC*. **B.** PCR analysis by agarose gel confirmed the genetic complementation. DNA fragments of the expected sizes of 925 bp and 1265 bp were amplified in the complemented strains B1940ΩprpB • pNLCT-*prpBC* and B1940ΩprpC • pNLCT-*prpBC*, respectively. **C.** Growth of *N. meningitidis* B1940, B1940ΩprpB, B1940ΩprpC and complemented strains (B1940ΩprpB • pNLCT-*prpBC* and B1940ΩprpC • pNLCT-*prpBC*) in MCDA-glucose (top) or MCDA-pyruvate

(bottom), in the absence or in the presence of 1 mM propionate. Values are means with standard deviations of two independent experiments as reported in File S2. Asterisks indicate statistically significant differences ($p < 0.05$) between *prpB*- or *prpC*-defective mutants and B1940, and between *prpB*- or *prpC*-defective mutants and *prpB*- or *prpC*-complemented strains.

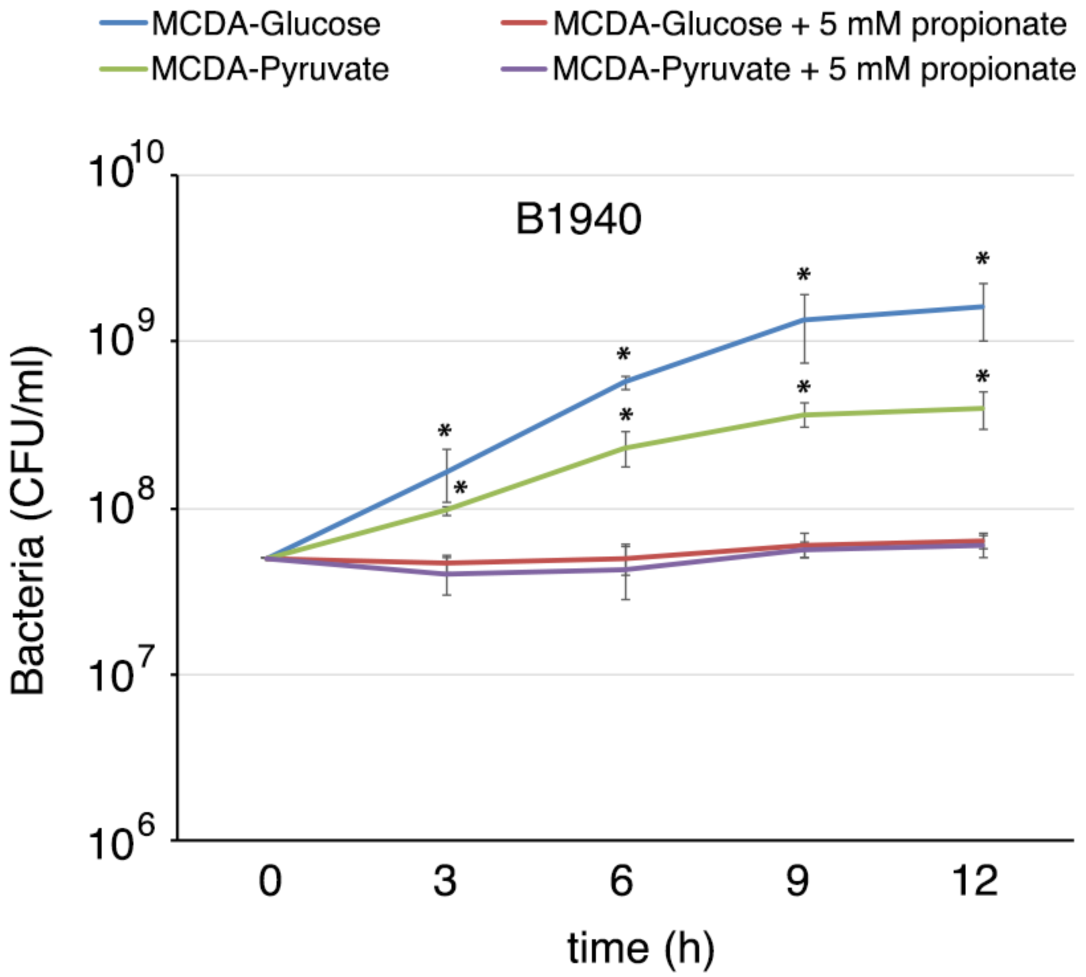

Fig. S3. Evaluation of colony forming units (CFU) of *N. meningitidis* B1940 grown in MCDA-glucose or MCDA-pyruvate in the absence or presence of 5 mM propionate. The data at each time point are the means and standard deviations of three independent experiments. Asterisks indicate statistically significant differences ($p < 0.05$) between bacteria grown in MCDA-glucose and MCDA-glucose + 5 mM propionate, and between bacteria grown in MCDA-pyruvate and MCDA-pyruvate + 5 mM propionate.

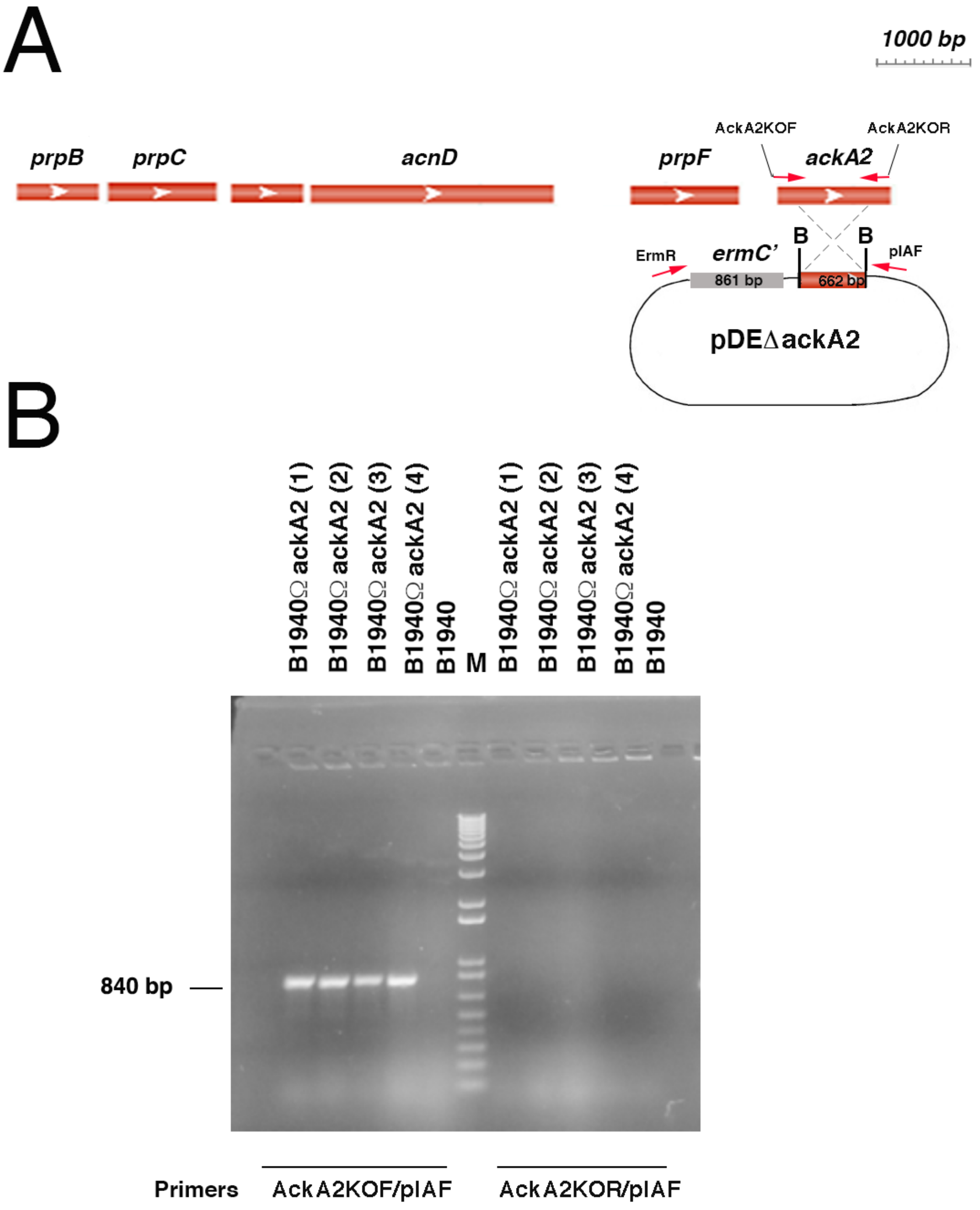

Fig. S4. Genetic inactivation of *ackA2* in *N. meningitidis* B1940. **A.** Map of the genomic region encompassing the MCC gene cluster in *N. meningitidis* MC58 and strategy for insertional inactivation of *ackA2.* **B.** PCR analysis by agarose gel confirmed the insertion of the erythromycin-resistant circular cassette by a single cross-over event in *ackA2*. For *ackA2* inactivation, four recombinant clones were obtained. As a result of the recombination event, a DNA fragment of the expected size of 840 bp was amplified in the transformed strains B1940ΩackA2(1), B1940ΩackA2(2), B1940ΩackA2(3) and B1940ΩackA2(4) using the primer pairs AckA2KOF/pIAF (shown in **A**).

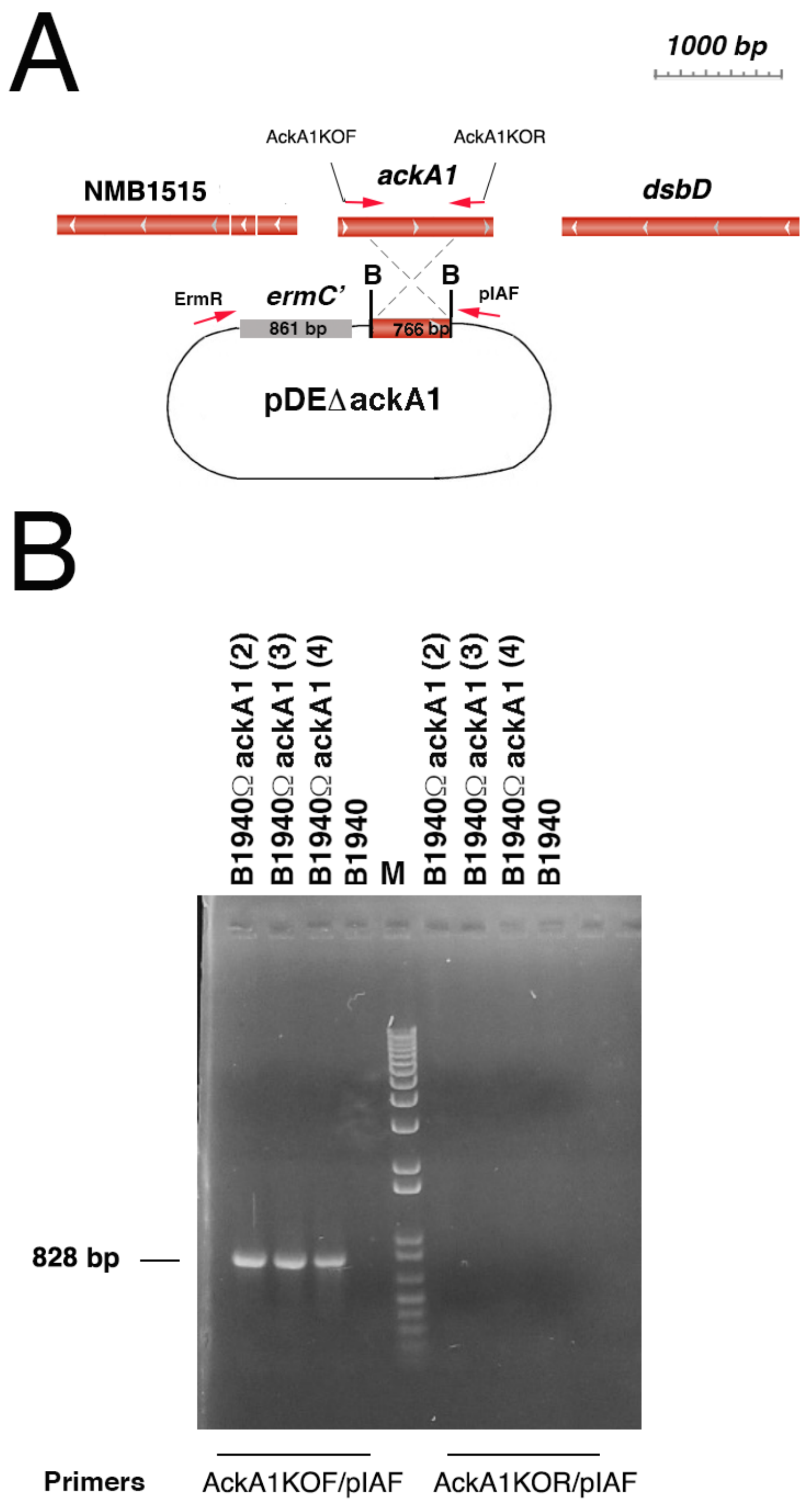

Fig. S5. Genetic inactivation of *ackA1* in *N. meningitidis* B1940. **A.** Map of the genomic region encompassing the MCC gene cluster in *N. meningitidis* MC58 and strategy for insertional inactivation of *ackA1*. **B.** PCR analysis by agarose gel confirmed the insertion of the erythromycin-resistant circular cassette by a single cross-over event in *ackA1*. For *ackA1* inactivation, three recombinant clones were obtained. As a result of the recombination event, a DNA fragment of the expected size of 828 bp was amplified in the transformed strains B1940ΩackA2(2), B1940ΩackA2(3), and B1940ΩackA2(4) using the primer pairs AckA1KOF/pIAF (shown in **A**).

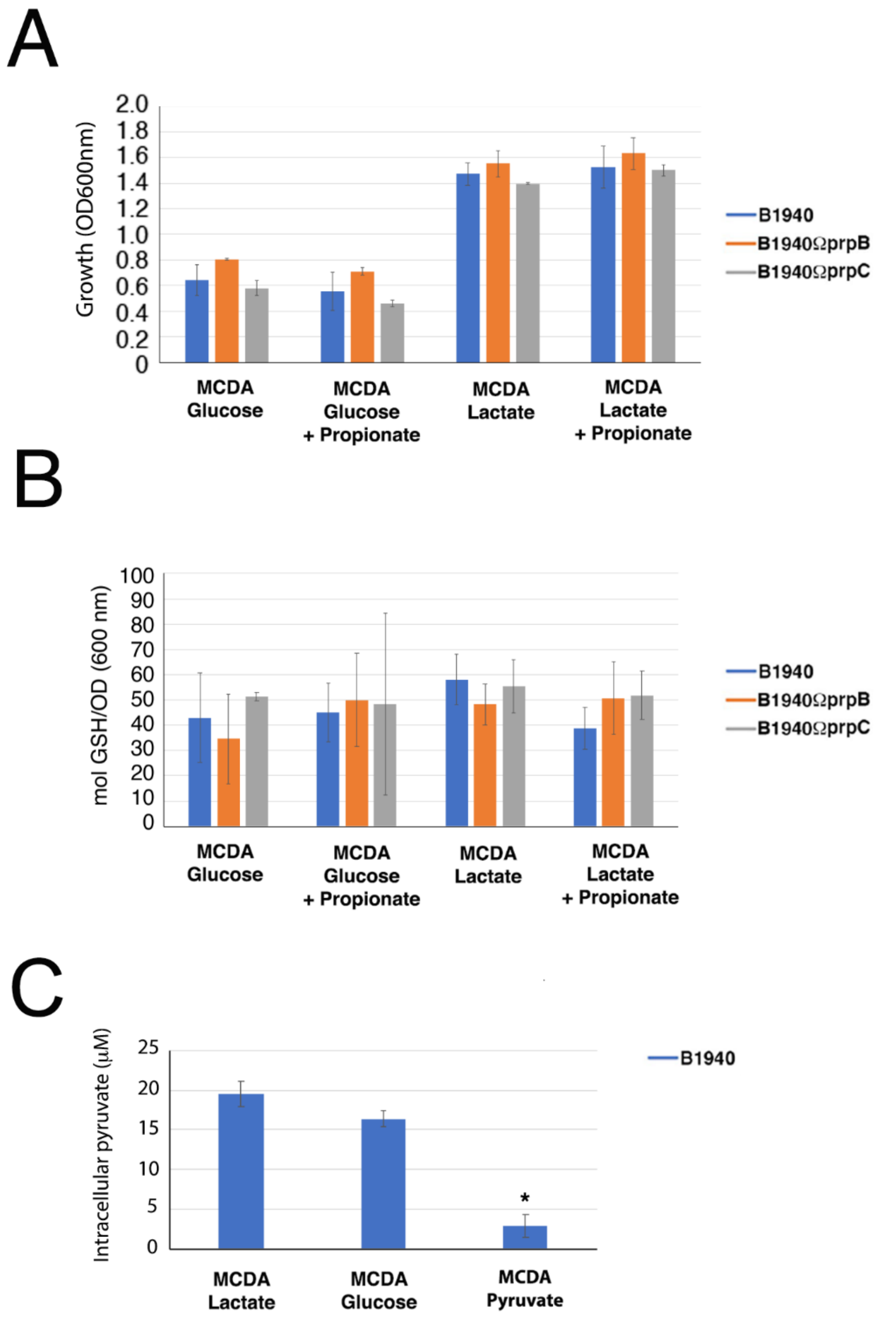

Fig. S6. Intracellular glutathione and pyruvate levels. **A.** *N. meningitidis* B1940 and isogenic *prpB*- and *prpC*-defective mutants were grown in MCDA-glucose or MCDA-lactate for 4 h. Then, cultures were supplemented with 5 mM propionate or left unsupplemented and growth was stopped 1 h later for determination of O.D. (600 nm) and intracellular glutathione levels. **B**. Determination of intracellular glutathione levels. The values indicate the means and standard deviations of triplicate sample values. **C.** Determination of intracellular pyruvate levels. The values indicate the means and standard deviations of triplicate sample values. Asterisk indicates statistically significant differences ($p < 0.05$) between the pyruvate concentration in MCDA-lactate, MCDA-glucose and MCDA-pyruvate.

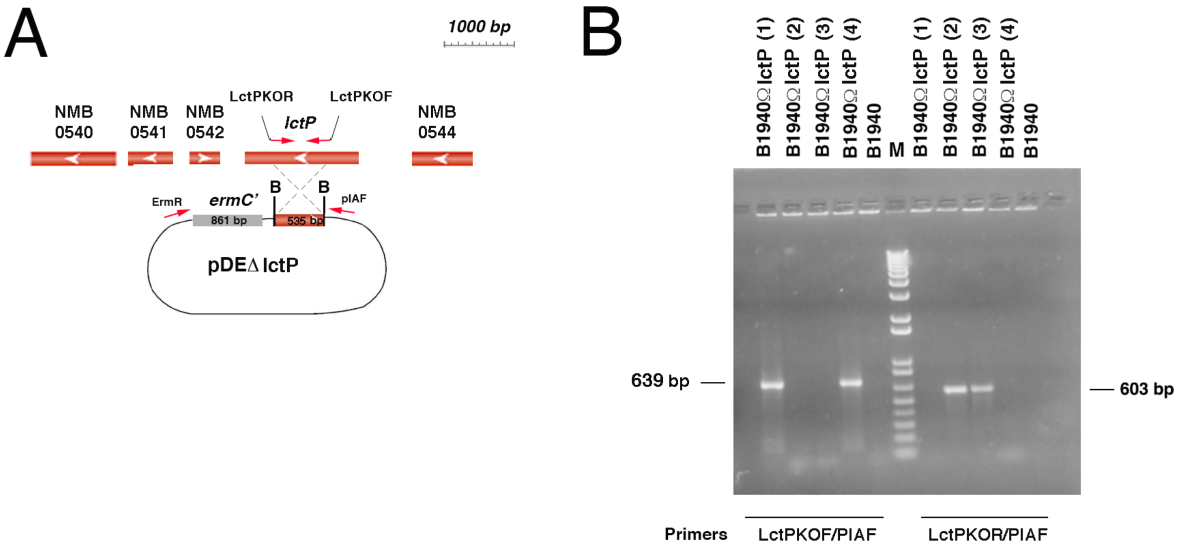

Fig. S7. Genetic inactivation of *lctP* in *N. meningitidis* B1940. **A.** Map of the genomic region encompassing *lctP* in *N. meningitidis* MC58 and strategy for insertional inactivation of *lctP*. **B.** PCR analysis by agarose gel confirmed the insertion of the erythromycin-resistant circular cassette by a single cross-over event in *lctP*. For *lctP* inactivation, four recombinant clones were obtained. As a result of the recombination event, a DNA fragment of the expected sizes of 639 bp was amplified in the transformed strains B1940ΩlctP(1) and B1940ΩlctP(4) using the primer pairs LctpKOF/pIAF (shown in **A**), and a DNA fragment of the expected sizes of 603 bp was amplified in the transformed strains B1940ΩlctP(2) and B1940ΩlctP(3) using the primer pairs LctpKOR/pIAF.

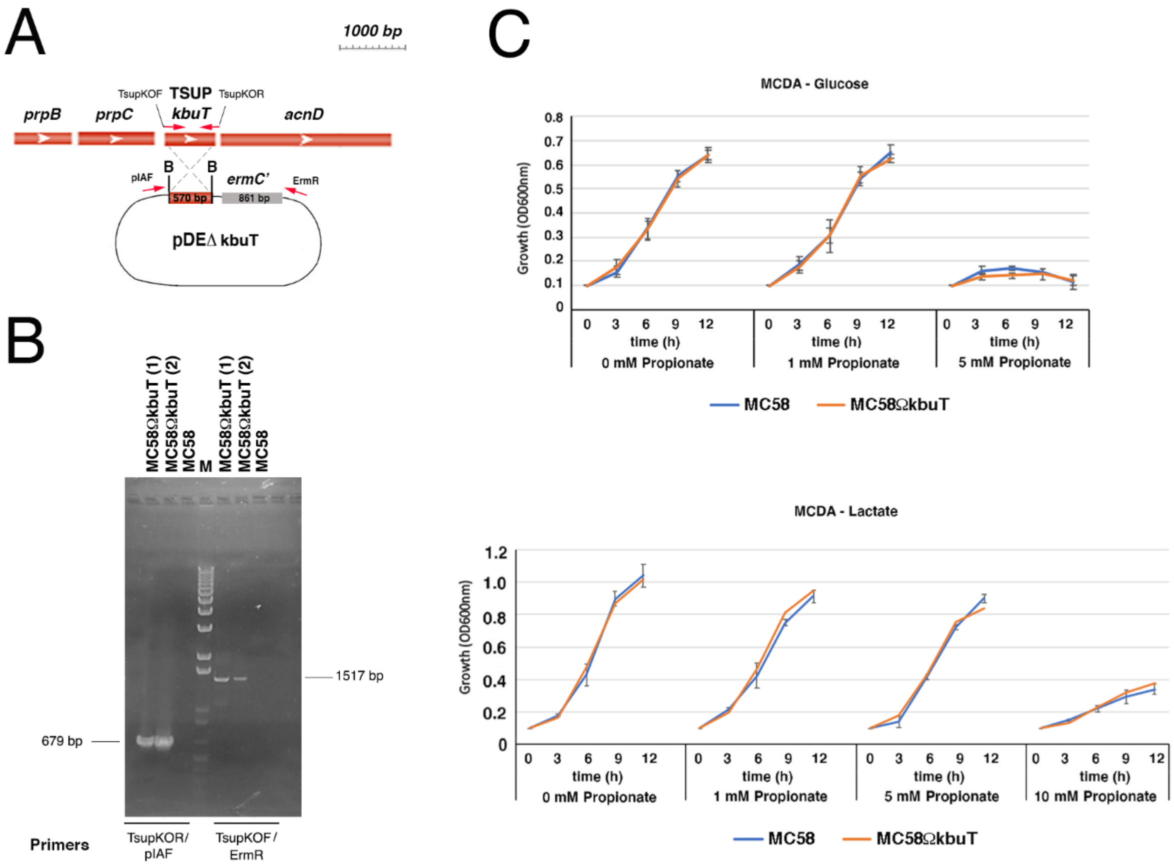

Fig. S8. Genetic inactivation of TSUP-encoding gene (*kbuT*) in *N. meningitidis* MC58 and growth curves of *N. meningitidis* MC58 and isogenic *kbuT*-defective mutant in MCDA medium with different carbon sources. **A.** Map of the genomic region encompassing the MCC gene cluster in *N. meningitidis* MC58 and strategy for insertional inactivation of the TSUP-encoding gene (*kbuT*). **B.** PCR analysis by agarose gel confirmed the insertion of the erythromycin-resistant circular cassette by a single cross-over event in *kbuT*. For *kbuT* inactivation, two recombinant clones were obtained. As a result of the recombination event, DNA fragments of the expected sizes of 679 and 1517 bp were amplified in the transformed strains MC58ΩkbuT(1) and MC58ΩkbuT(2) strains using the primer pairs TsupKOR/pIAF and TsupKOF/ErmR, respectively (shown in **A**), according to the orientation of the fragment in the shuttle vector pDEX. **C.** Growth of *N. meningitidis* MC58 and *kbuT*-defective mutant in MCDA-glucose (upper panel) or MCDA-lactate (lower panel), in the absence or in the presence of different concentrations of propionate. Two independent *kbuT*-defective mutants (1, 2) were tested in three independent experiments (File S5). Means and standard deviations are shown at each time point.

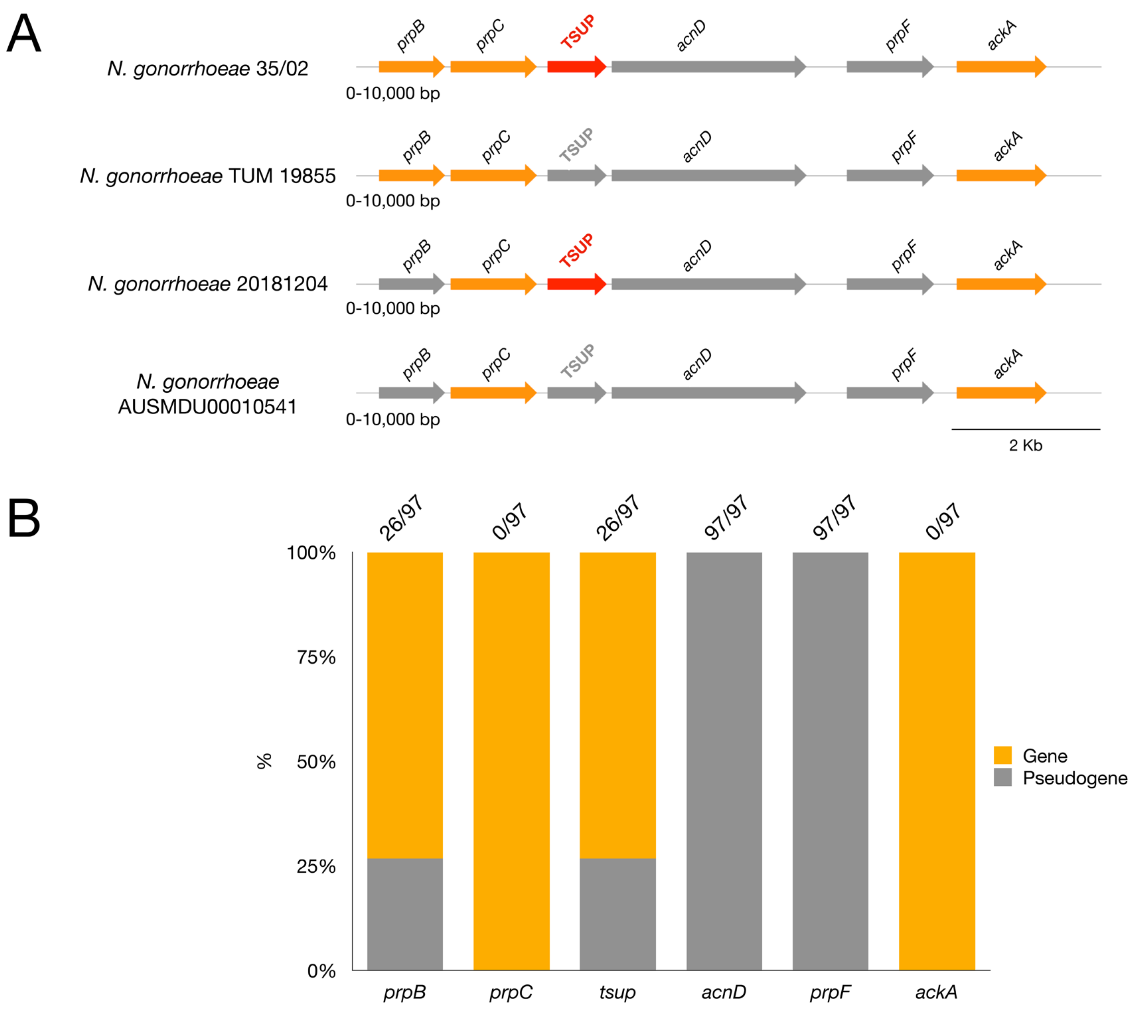

Fig. S9. Organization and variation of the MCC gene cluster locus in *Neisseria gonorrhoeae* and other *Neisseria* spp. **A.** Genetic map of the MCC gene cluster in completely annotated genomes of *Neisseria gonorrhoeae* strains. **B.** Co-occurrence of synthetic genes or pseudogenes with *kbuT* gene or pseudogenes in completely annotated genomes of *Neisseria gonorrhoeae* strains.

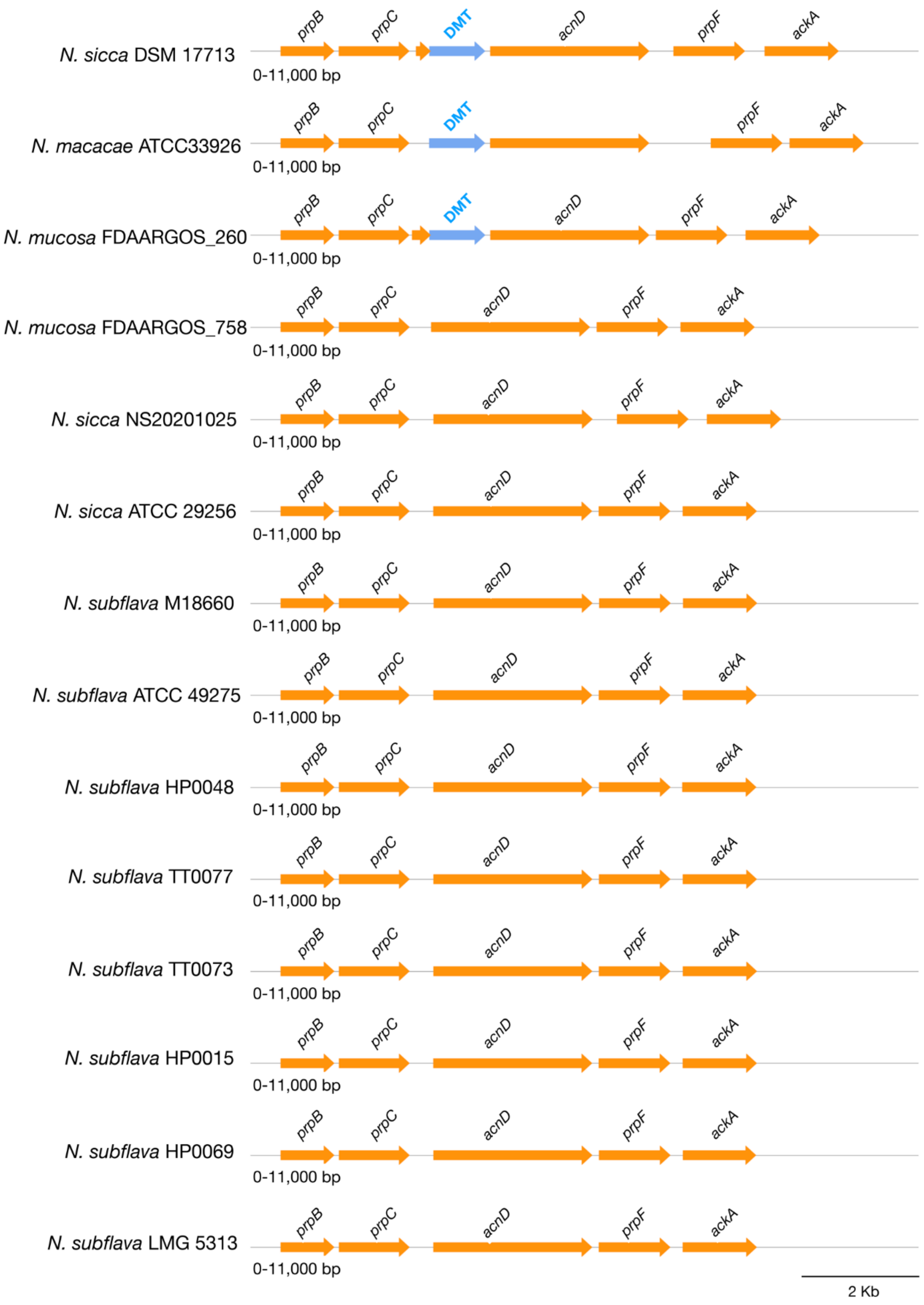

Fig. S10. Maps of the genomic regions between *prpC* and *acnD* in non-pathogenic *Neisseria* spp.

Supplementary Files

File S1. Primary data of growth curves of *N. meningitidis* B1940 and isogenic *prpB*- and *prpC*-defective mutants in MCDA-glucose, MCDA-pyruvate or MCDA-lactate, in the absence or in the presence of different concentrations of propionate. Three independent *prpB* mutants were tested (1, 2, 3), while a single *prpC* mutant (1) was obtained and analyzed in triplicate experiments. Means and standard deviations are shown at each time point. The file also contains p-values for assessing statistical significance. $p < 0.05$ are marked in red.

File. S2. Primary data of growth curves of *N. meningitidis* B1940, B1940ΩprpB, B1940ΩprpC and complemented strains (B1940ΩprpB • pNLCT-*prpBC* and B1940ΩprpC • pNLCT-*prpBC*) in MCDA-glucose or MCDA-pyruvate, in the absence or in the presence of 1 mM propionate. The file also contains p-values for assessing statistical significance. $p < 0.05$ are marked in red.

File S3. Primary data of growth curves of *N. meningitidis* B1940 and isogenic *ackA1*- and *ackA2*-defective mutants in MCDA-glucose, in the absence or in the presence of different concentrations of propionate. Three independent *ackA1*-defective mutants (2, 3, 4) and two independent *ackA2*-defective mutant (1, 2) were tested in independent experiments. Means and standard deviations are shown at each time point. The file also contains p-values for assessing statistical significance. $p < 0.05$ are marked in red.

File S4. Primary data of growth curves of *N. meningitidis* B1940 and isogenic *lstP*- defective mutants in MCDA-glucose, MCDA-pyruvate or MCDA-lactate, in the absence or in the presence of different concentrations of propionate. Two independent mutants for *lstP* (1, 2) were tested, along with the wild type B1940, in independent experiments. Means and standard deviations are shown at each time point. The file also contains p-values for assessing statistical significance. $p < 0.05$ are marked in red.

File S5. Primary data of growth curves of *N. meningitidis* MC58 and isogenic *kbuT*-defective mutants in MCDA-glucose or MCDA-lactate, in the absence or in the presence of different concentrations of propionate. Two independent *kbuT*-defective mutants (1, 2) were tested in three independent experiments. Means and standard deviations are shown at each time point. The file also contains p-values for assessing statistical significance. $p < 0.05$ are marked in red.

File S6. Primary data analysis of *N. meningitidis* strains with BIOLOG Phenotype MicroArray™ system. Two independent *prpB* (1, 2) and *kbuT*-defective mutants (1, 2) were tested, together with the single *prpC* mutant obtained (1), the wild-type *N. meningitidis* strains B1940 and MC58, and the *N. lactamica* strains 995 and 411.

Supplementary Table

Table S1. MCDA vs. CDM composition (Catenazzi et al., 2014).

**MCDA (Pagliarulo et al., 2004)**		**CDM (Catenazzi et al., 2014)**	
**Chemical**	**Final conc.**		
NaCl	100 mM	NaCl	100 mM
KCl	2.5 mM	KCl	-
K_2SO_4	-	K_2SO_4	5.75 mM
NH_4Cl	7.5 mM	NH_4Cl	18 mM
Na_2HPO_4	7.5 mM	Na_2HPO_4	-
KH_2PO_4	1.25 mM	KH_2PO_4	23 mM
$Na_3C_6H_5O_7 \cdot 2H_2O$	2.2 mM	$Na_3C_6H_5O_7 \cdot 2H_2O$	-
$MgCl_2$	-	$MgCl_2$	1.95 mM
$MgSO_4 \cdot 7H_2O$	2.5 mM	$MgSO_4 \cdot 7H_2O$	-
$MnSO_4$	0.0075 mM	$MnSO_4$	-
L-glutamic acid	8.0 mM	L-glutamic acid	-
L-glutamine	-	L-glutamine	4 mM
L-arginine	0.5 mM	L-arginine	0.7 mM
Glycine	2.0 mM	Glycine	3.8 mM
L-serine	0.2 mM	L-serine	4.75 mM
L-cystine	-	L-cystine	3.8 mM
L-cysteine$\cdot$HCl$\cdot H_2O$	0.06 mM	L-cysteine$\cdot$HCl$\cdot H_2O$	-
Glycerine	0.5% (v/v)	Glycerine	-
$CaCl_2 \cdot 2H_2O$	0.25 mM	$CaCl_2 \cdot 2H_2O$	0.20 mM
Ferric citrate	-	Ferric citrate	0.15 mM
$Fe_2(SO_4)_3$	0.01 mM	$Fe_2(SO_4)_3$	-
$NaHCO_3$	-	$NaHCO_3$	10 mM
Glucose	16,5 mM (0,3%)	Glucose	2.5 mM
Na pyruvate	10 mM	Na pyruvate	5 mM
Na lactate	26,7 mM (0,3%)		
Propionic acid	-	Propionic acid	5 mM
Na propionate	0 - 10 mM	Na propionate	-
pH	7.4	pH	7.0
